# Feelings of Polish and Palestinian Students after Receiving Vaccinations against COVID-19

**DOI:** 10.3390/ijerph192013692

**Published:** 2022-10-21

**Authors:** Krzysztof Zdziarski, Katarzyna Karakiewicz-Krawczyk, Mariam S. Awad, Narmeen Qumsieh, Anna Landowska, Beata Karakiewicz

**Affiliations:** 1 Subdepartment of Social Medicine and Public Health, Department of Social Medicine, Pomeranian Medical University in Szczecin, 71-210 Szczecin, Poland; 2 Department of Specialist Nursing, Pomeranian Medical University in Szczecin, 71-210 Szczecin, Poland; 3 Faculty of Nursing and Health Sciences, Bethlehem University, Bethlehem 92248, Palestine; 4 Faculty of Economics, Finance and Management, University of Szczecin, 71-101 Szczecin, Poland

**Keywords:** vaccine, COVID-19, student, feelings, Palestine, Poland

## Abstract

The COVID-19 pandemic has limited human freedom in many areas. Developing a COVID-19 vaccine has been a key task to contain the spread of the virus. In many countries, there is increasing concern about anti-vaccines due to complications after receiving the vaccine. The research problem concerns the opinions of Polish and Palestinian students after receiving vaccinations against COVID-19. This study involved 657 respondents (332 from Poland and 325 from Palestine) who completed the original questionnaire online. The respondents present two different cultures, embedded in different existential conditions, also in terms of health care, and especially the availability of vaccines. The obtained data indicate that almost 50% of research participants from both countries believe that vaccines are an effective antidote to the pandemic situation. Respondents in both populations believed that it was their personal choice to undergo vaccinations. The social motivation for vaccination in both groups was the desire to participate in public life, and the possibility of free travel for Poles, and the fear of infecting other people for Palestinians. The most common side effect reported after vaccination was pain at the site of the infection. Medical assistance was more often sought by respondents from Palestine. From an existential, psychosocial and health perspective, vaccines contributed to strengthening the vital forces in a large part of the population, allowed rebuilding social interactions and gave a sense of security in the daily functioning of a person.

## 1. Introduction

The COVID-19 pandemic has produced enormous health and psychosocial imbalances in human existence all over the world. The scale of fear of a pandemic increased and caused stress, anxiety, and it generated the use of narcotic drugs in some social groups to reduce emotional tension [1]. Additionally, medical personnel in many cases were paralyzed with fear and even suicidal thoughts [2]. The destruction affected not only medical workers but also students, who, physically isolated, were unable to cope with the nihilistic emotional state [3,4]. Following the adoption of vaccines, some scientists believe that the virus no longer poses a threat to human health. According to other researchers, the next wave of the pandemic is expected in the fall. The results of medical and social studies show that the administered vaccines and the maintenance of epidemic restrictions have significantly contributed to stopping the pandemic. The research undertaken, covering almost all areas of life, allowed identifying the factors that had the greatest impact on the decision to take the vaccine by individual social groups. Based on the data obtained from the review of articles indexed in reputable databases (Pub Med (MEDLINE), Elsevier, Science Direct, and Scopus), it is possible to identify the factors that decided whether to undergo vaccination. These are safety, effectiveness, trust, social impact, fear and anxiety [5]. Scientists are still looking for factors that reflect authentic opinions after taking vaccinations. The wealth of data collected in this space presents a picture of the reality perceived by various groups of people along with their problems. The broad context of the research on the consequences of the COVID-19 pandemic shows many existential and health problems not only for the elderly, but also for children and adolescents. For example, anxiety and depressive behavior are factors that scientists believe may result from disrupted education and the negative impact of the pandemic on the existential well-being of families due to isolation (pandemic and political), limited access to health care, and lack of material resources for everyday life, especially of families with already low income [6]. Other reports show that low- and middle-income countries are unable to secure immunization against COVID-19 [7]. Research also shows dissonance in the approach to vaccination of different nationalities. For example, the inhabitants of Saudi Arabia are concerned about the adverse effects of vaccinations, although they are not life threatening, and believe that unvaccinated people should not have the right to a full public life [8,9]. In contrast, Italian respondents showed that information on vaccination is not sufficient for people with low levels of education, and for inhabitants of the islands and southern regions of the country [10]. Considering comorbidities, it is worth recalling the results of studies from Japan, where 90% of patients with enteritis disease, after being educated about the effectiveness of the COVID-19 vaccine, decided to adopt it [11].

At the start of the pandemic, low COVID-19 vaccine acceptance rates were recorded in the Middle East, Russia, Africa, and several European countries. This fact has been a serious problem in global efforts to contain the pandemic, so more studies are recommended to convince the reluctant and undetermined [12]. It should be added that social opinions after taking vaccines change due to the occurring various health complications attributed to vaccines. However, the results of studies conducted in many regions of the world more often indicate a positive attitude towards the administered vaccines, also in patients with comorbidities [13,14]. Some countries also see a positive attitude towards influenza vaccination with concomitant administration of the COVID-19 vaccine [15]. Because of the vaccination against COVID-19, many emotions are aroused by vaccinating children whose parents do not fully accept the proposed vaccines, emphasizing that they have not been adequately tested and there is no information about side effects [16,17].

The aim of this study was to check the opinions of Polish and Palestinian students as to the effectiveness of vaccinations against COVID-19. Both populations live in different geographic zones and social, health and political conditions. For this reason, it is meaningful to learn about the opinions of young people in terms of the research problem undertaken. This study shows the vital role of the access time to a vaccine and the resulting opinion of young people who are often critical of vaccination. It should be emphasized that Palestinian students started receiving vaccinations later than other communities, which was caused by the lack of availability of vaccines due to cross-border conditions and, consequently, by limited epidemiological activities [18]. The undertaken problem of research among students is part of the general discussion on the adopted vaccinations, where universities play a huge role in organizing vaccinations at university campuses, as well as in generating pro-vaccination attitudes [19].

## 2. Materials and Methods

This study has been conducted using the author’s survey in March 2022 among students of universities in Poland and Palestine. The questionnaire in addition to socio-code data contained the following questions:

1. What type of vaccine did you receive?
2. What dose of the vaccine did you receive?
3. Who influenced your decision to vaccinate against COVID-19?
4. What was your motivation for taking the vaccine?
5. Please describe what undesirable effects occurred in your case after 1, 2, 3 vaccination.
6. Did you feel fear while receiving the vaccine?
7. Was a doctor’s help needed after receiving the vaccine?
8. Were you hospitalized after receiving the vaccine?
9. Are you satisfied with receiving the vaccine?
10. Are you worried about the negative effects of receiving the vaccine?
11. Has any of your loved ones had COVID-19 infection?
12. Are you afraid of infection and falling ill with COVID-19 by someone close to you?
13. Did any of your loved ones die from COVID-19?
14. How do you rate the effectiveness of the vaccine you have received?
15. Do you think the adoption of the vaccine will help to contain the pandemic?
16. Do you observe the following epidemic restrictions?

The research was conducted at the University of Bethlehem, Palestine and the Pomeranian Medical University in Szczecin, Poland. The respondents filled in the original online questionnaire. The study participants were invited to this study on purpose because universities represent two different cultures, which allows the COVID-19 vaccination problem to be viewed from a variety of interesting perspectives. Moreover, different health care systems draw an interesting perspective on the approach to a patient who in Palestine is embedded in a space of constant military conflict and lives in conscious or less conscious existential stress, fear of a pandemic, and has had limited access to vaccines for some time, and lives in Poland in peaceful conditions and has free access to vaccinations [20].

The health care system in Palestine is caring but constantly strives to provide adequate medical services. The health system does not have sufficient infrastructure; there is a shortage of medical equipment, medicine and protective equipment. The ongoing conflict led to the ruin of many hospitals and clinics. Health care work is hampered by a shortage of electricity and water is scarce in some regions. Palestine suffers from a shortage of family medicine, pediatrics, psychiatry, neurology, oncology, and pediatric surgery suffer from serious staff shortages. In the West Bank and the Gaza Strip, medical care for refugees faces big problems. Movement restrictions make access to medical services extremely difficult [21]. Political and humanitarian conditions in Palestine are complex and have a large impact on the health care system (usually for a fee), especially in terms of epidemic prevention, including COVID-19. The difficult humanitarian situation in Palestine, including poor socio-economic and living conditions, and an inefficient health care system, greatly hampered the ability to respond to COVID-19. The long-standing political instability and geographical separation between the Gaza Strip and the West Bank generate new existential challenges and create poorer humanitarian conditions during a pandemic. Still, unmet humanitarian needs increased the risk of spreading COVID-19, especially among vulnerable groups, including Palestinian refugees, often living in overcrowded camps, people from poor socio-economic backgrounds, prisoners, and patients in need of urgent medical treatment, especially pregnant women [22]. The researchers point out that the Palestinian pandemic was exacerbated by poor health infrastructure. The Palestinian Ministry of Health (PMoH) has done everything in its power with limited resources. However, the political context, Palestinian authority, jurisdiction and border control were factors that blocked the delivery of test materials and supportive measures for the care of COVID-19 patients [23]. In the Polish state health system, care for COVID patients was carried out at European standards. Access to vaccinations, medical care, and hospital stays were the usual tools during the COVID-19 pandemic. Poland had disruptions in the provision of health services as in other European countries. Research from 2021 shows that with the generally good condition of primary health care (POZ), almost 50% of Poles reported barriers in accessing health services in the last 12 months. The most frequently indicated were long waiting times and temporary closure of health care facilities [24]. It should be added that primary health care (POZ) provides comprehensive and coordinated health care services at the place of residence to all eligible persons residing/staying in the territory of Poland. Services are provided on an outpatient basis in a clinic, and in medically justified cases, also at the patient’s home [25]. In view of the above, it should be noted that the data collected in the study represent two different areas in the pandemic space. The captured feelings after receiving vaccinations are valuable in the discussion on the general health condition of populations in constant existential threat due to armed conflicts.

The research group had 657 people, including 332 respondents from Poland and 325 from Palestine. The answer indicator was 100%. In the group of Polish students, women accounted for 76.5%, men 23.5%. However, among the respondents from Palestine, women accounted for 79%, and men 21%. The average age of respondents from Poland and Palestine is 22.43 and 19.71, respectively. Considering the age of respondents due to gender, women from Poland and Palestine had an average age of 22.72 and 19.58, and men 21.5 and 20.24 years. Undergraduate or engineering studies from Poland constituted 30.4% of the total Polish students, the second degree (masters) 69%, and the third degree (doctoral) 0.6%. In the group of students from Palestine, students of first-cycle studies were 99.4%, the remaining 0.6% are master’s students. Most students from Poland are students of physiotherapy 30.7%, cosmetology 19.9%, dietetics 19%, and nursing 10.8%.

The most numerous group of respondents from Palestine are from the Faculty of Nursing and Health Sciences 59.1%, the Faculty of Business Administration 11.7%, the Faculty of Arts 10.5%, the Faculty of Education 9.2%, and the Faculty of Science 6.2%. Considering the place of residence of the respondents, 59% of Polish students live in a large city (over 100,000 inhabitants), 24.1% in a small city (up to 100,000 residents), and the remaining 16.9% of respondents live in the countryside. A total of 41.8% of Palestinian respondents live in the countryside, 35.7% in a large city, 22.5% in a small city. Students have adopted various types of vaccines. Most students from Poland and Palestine have adopted a Pfizer vaccine, 83% (PL) and 78% (PAL), respectively. Respondents from Poland have also adopted Johnson & Johnson 8%, AstraZeneca 7% and Moderna 2%. Among students from Palestine, the other students adopted Sputnik 16% and Moderna 6%. Table 1 shows the quantitative characteristics and average and standard deviation of the age of people taking part in the survey (nationality, gender and age).

## 3. Results

The quantitative data were obtained from the survey in the form of average and standard deviation as well as in the form of percentages and graphically with histograms. A chi-square test and the Mann–Whitney test were used to analyze the differences between the decks. The significance level of 0.05 was adopted for all tests. To measure the credibility of questions with the answers given according to the Likert scale. Cronbach’s alpha was used.

Analyzing the opinions of the respondents regarding the efficiency of the adopted vaccine, the results of the chi-square test indicate that the answer to this question depends on the students’ nationality (*p* < 0.001). Data indicate that 25.5% of Palestine students do not believe in the effectiveness of the vaccine adopted, and this percentage is 9.3% among students from Poland. In both groups, less than 50% of respondents recognize the vaccine as effective. Comparing the results of the answer to this question due to gender, there were no statistically significant differences in the responses between women and men.

Considering the answer to the question regarding the fear of negative effects after the vaccine adoption, the results of the group of students from Poland and Palestine differ significantly statistically (*p* < 0.001). Over half of the students from Palestine (57.8%) are afraid of negative effects of vaccination, in the case of Polish students this percentage is 31.9%. A total of 52.1% of the respondents from Poland and 34.2% from Palestine are not afraid of side effects. Similarly, statistically significant differences on this question were found between women and men from Poland and Palestine (*p* < 0.001). Data indicate that 63% of women from Palestine and 38.2% from Poland are afraid of side effects after vaccination. Despite this, over 50% of women are satisfied with the part of the vaccine. It should be added that 66.7% of men from Poland and 34.2% from Palestine are not afraid of negative effects of vaccination. The chi-square test also showed statistically significant differences between the answers of Polish and Palestinian students to the question about satisfaction from the adopted vaccine. Over half of the respondents from both groups are satisfied with the fact that they are vaccinated, but 24% of the respondents from Palestine and 8.7% of people from Poland have no such feeling. Analyzing satisfaction from vaccination due to the gender of two groups of respondents, there were no statistically significant differences (*p* > 0.05).

Using the chi-square test, it has been shown that the answers to the question if the acceptance of the vaccine will stop the pandemic depend on the students’ nationality (*p* < 0.01). It agrees with this statement of 23.8% of students from Poland and 11.1% from Palestine. A total of 43.7% of respondents from Poland and Palestine agree or rather agree. However, 16% of students from Palestine and 10.2% from Poland believe that vaccination will not contribute to stopping the pandemic. The chi-square test showed that there are no significant differences in the answer to this question due to the gender of respondents. In addition, the respondents were asked who had an impact on the decision to adopt a vaccine. The chi-square test showed statistically significant differences in the replies of respondents from Poland and Palestine. Over 80% of the respondents from Poland declared that the acceptance of the vaccine was their personal, independent decision. Among the Palestinian respondents, this percentage was 59.7%. Information that the family decided about the vaccination was reported by 6.9% of respondents from Poland and 16% from Palestine.

**Figure 1:**
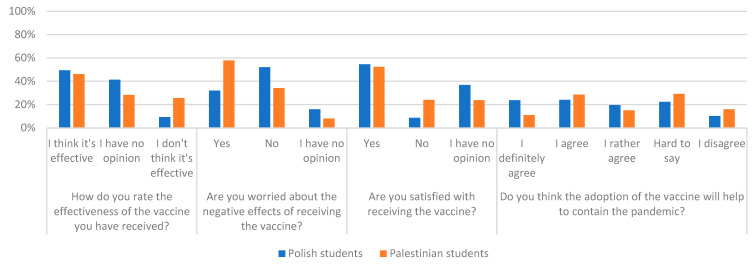
Percentage of answers to individual questions due to the nationality of respondents.

**Figure 2:**
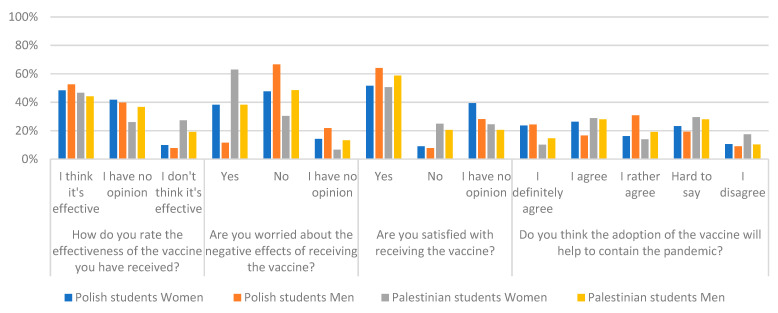
Percentage of the answers of two groups of respondents to individual questions due to gender and nationality.

There were significant statically differences in answers to questions about the threat to the pandemic of nearby surveyed persons. The question whether someone on COVID-19 suffered among your relatives, confirmed 87.7% of Polish respondents and 76.3% of Palestinian respondents. The question about death because of COVID-19 a loved one, 28% from Palestine and 14.2% from Poland confirmed such an event. Detailed quantitative data and chi-square test results for the answers to the above questions broken down into nationality and gender respondents are presented in Table 2 and Table 3 and graphically in Figure 1 and Figure 2.

**Figure 3:**
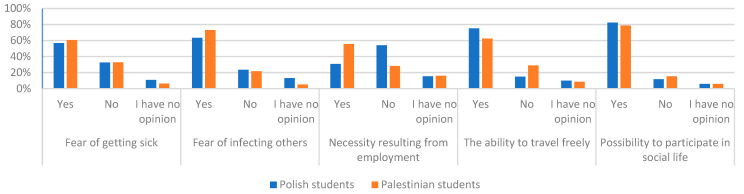
Percentage of answers to questions about the motivation of respondents to accept the vaccine.

Polish and Palestinian students also answered the question, which motivated them to adopt a vaccine. Most people, approximately 80% of two groups of respondents, admitted that possibilities to participate in social life (meetings, parties, going to a restaurant/pub, cinema, theater) were meaningful. The second most common factor that motivated Polish respondents (75%) to take the vaccination was the ability to travel freely, while Palestinian students (72.9%) was fear of infecting others. For three answers, the chi-square test showed dependence on nationalities. They were fere of infecting others, necessity resulting from employment and the abilities to travel freely. The least often respondents from Poland and Palestine as a motivating factor for vaccination indicated the necessity of resulting from employment. Palestinian students more often (55.7% of people) than Polish students (30.7% of people) pointed to the necessity resulting from employment as an incentive for vaccination. The chi-square test did not show differences in the fear of getting sick factor as motivating to vaccinations. A total of 56.6% of students from Poland and 60.6% from Palestine pointed out that this factor motivates them to vaccinate. Response results to the motivation to adopt the vaccine are presented in Figure 3.

**Figure 4:**
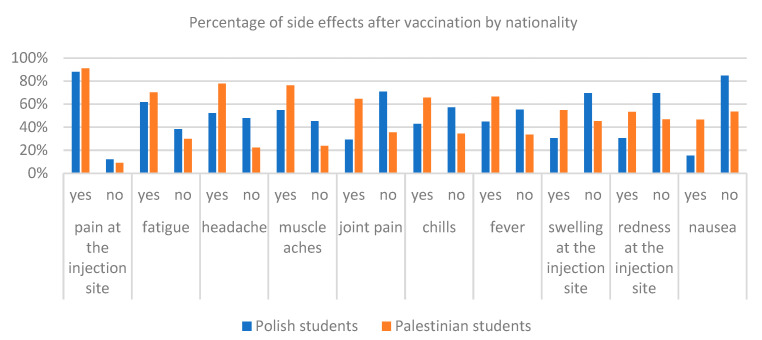
Comparison of the number of side effects after taking the vaccine by nationality.

Analyzing responses to the question about the occurrence of side effects after the vaccine adoption, the chi-square test showed that in addition to pain in the injection site, in all other cases of side effects, their appearance is dependent on the respondents’ nationality (*p* < 0.001). Pain at the place of administration of the vaccine occurred in 88% of the respondents from Poland and 91% from Palestine. Statistically, these results are not dependent on the nationality of respondents (*p* = 0.191). In all other answers to the question of the occurrence of side effect, the respondents from Palestine significantly confirmed the instance (*p* < 0.001). The largest differences between Polish and Palestinian students were observed in question about the occurrence of joint pain. As many as 5% of Palestine respondents confirmed this effect compared to 29% of respondents from Poland. Itching at the place of injection confirmed 55% of Palestinian and 30% of Polish respondents. Detailed quantitative results and the chi-square tests of respondents from Poland and Palestine on the occurrence of side effects are presented in Table 4 and graphically on Figure 4.

**Figure 5:**
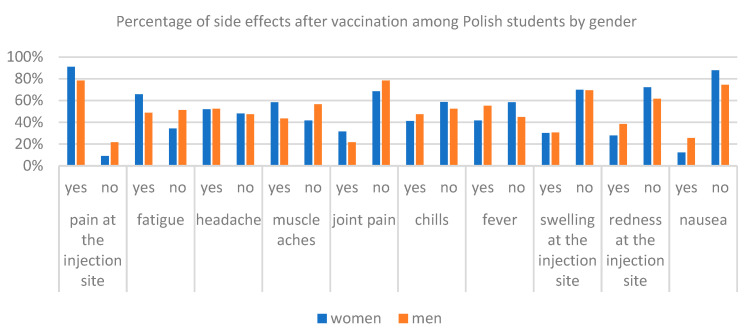
Comparison of the number of side effects after receiving the vaccine among Polish students by gender.

**Figure 6:**
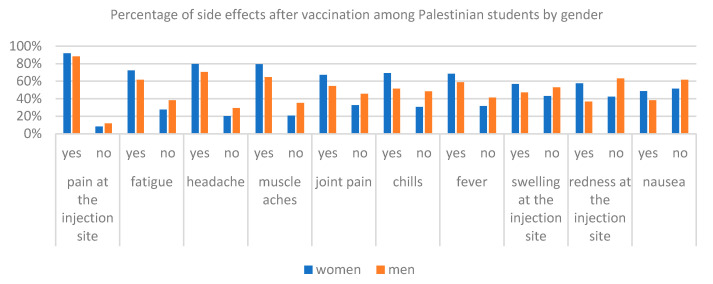
Comparison of the number of side effects after receiving the vaccine among Palestinian students by gender.

With the help of the chi-square test, statistically significant differences between the answers were examined regarding the occurrence of side effects among Polish and Palestinian respondents due to gender (Table 4, Figure 5 and Figure 6). Among Polish respondents answers to questions about pain in the injection site, fatigue, muscle pain, fever and nausea after vaccination are dependent on gender. In this case, as many as 91% of the respondents of women and 78% of men from Poland felt pain in the place of injection, 66% of women and 49% of men experienced fatigue, 58% of women and 44% of men experienced muscle pain, 42% of women and 55% of men had fever and 12% of women and 26% of men from Poland felt nausea after vaccination. Differences in Palestinian responses to the question about the side effects of the chi-square test showed for answering questions about headaches, muscle pain, joint pain, chills and redness in the place of the evolution. In all these five cases, more women have stated such a side effect of vaccination than men. For example, headaches was indicated by 80% of women from Palestine and 71% of men, muscle pain was indicated by 79% of women and 65% of men, joint pain was indicated by 67% of women and 54% of men, chills were indicated by 69% of women and 51% of men, and redness was indicated 58% of women and 37% of men from Palestine. Percentages of answers of respondents from Poland and Palestine for questions about the side effects are presented according to Figure 4 and Figure 6.

**Figure 7:**
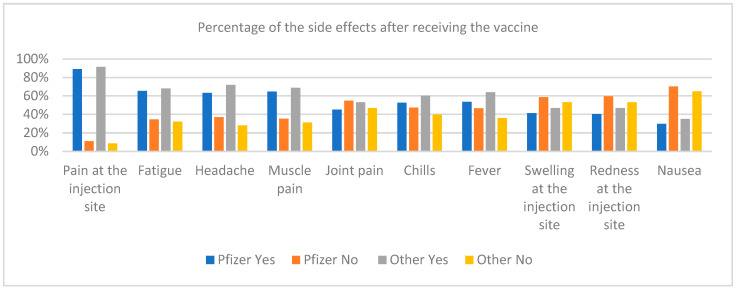
Percentage of the occurrence of side effects after vaccination with Pfizer and other vaccines (i.e., Moderna, AstraZeneca, Johnson & Johnson, and Sputnik).

To analyze the occurrence of side effects after vaccination with Pfizer or other vaccines (Moderna, AstraZeneca, Johnson & Johnson and Sputnik), for the group of respondents from Poland and Palestine, a chi-square test was performed. This test showed that there is a statistically significant difference for the “fever” side effect. It can be concluded that the fever has occurred frequently after vaccination with other vaccines than with Pfizer. For other types of post-vaccination side effects, no statistically significant differences were found in their occurrence depending on the type of vaccine. Figure 7 shows the percentage of the occurrence of a side effect after vaccination in the number of vaccinations with Pfizer or other vaccine.

**Figure 8:**
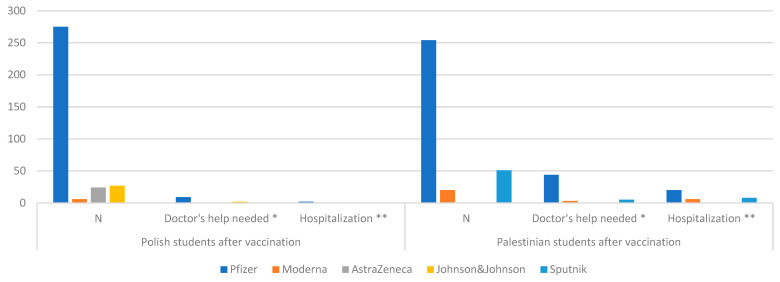
Comparison of the number of Polish and Palestinian students needing a doctor’s help or hospitalized after vaccination. N number of vaccination; * each person is counted only once if there was a need for a doctor’s help, regardless of the number of contacts and vaccination doses, excluding hospitalizations; ** each person is counted only once if there is a need for hospitalization, regardless of the number of hospitalizations and vaccination doses.

When considering unwanted individual effects, the respondents also answered the question if a doctor’s help was needed after taking the vaccine and if the respondent was hospitalized. In a group of 325 students from Palestine as many as 52 people (16%) stated that they required a doctor’s help, while 34 students were hospitalized (10.5%). In the group of Polish students, this percentage was lower. Among the 332 vaccinated people, 11 people (3.3%) stated in the survey that they required help and 2 people (0.6%) were hospitalized; see Figure 8.

In addition, they were asked if they are afraid of infection and becoming ill on COVID-19 and whether the same fears feel in relation to their relatives (Table 5). Answers were obtained on a scale from 1 (no concern) to 5 (strong fears). Cronbach’s alpha coefficient for questions regarding Polish and Palestinian students is 0.73 and 0.72; therefore, general credibility of the questionnaire was achieved. Among Polish and Palestinian students, fears of infecting and disease themselves are above 2.5. The results obtained do not show statistically significant differences in these two groups (*p* = 0.17). On the other hand, fears for infection and disease of a loved one are stronger among the respondents from Poland. The average amounted to 3.92. The average score for Palestinian respondents is 3.1. The Mann–Whitney test indicates a statistically significant difference in Palestinians and Poles (*p* < 0.001).

**Figure 9:**
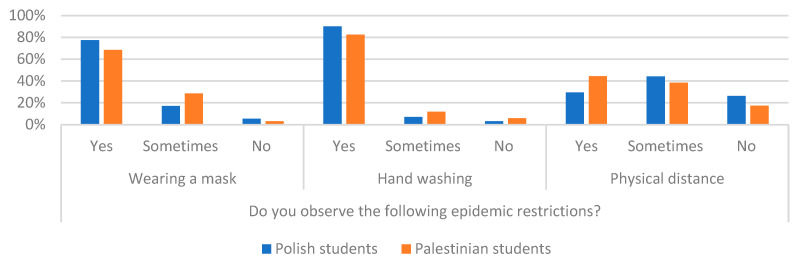
Percentage of answers to questions about compliance with epidemic restrictions of Polish and Palestinian respondents.

Students from Poland and Palestine were asked if they observe epidemic restrictions (wearing a mask, hand washing, and distancing behavior). The chi-square test showed statistically significant differences in three cases by comparing the answers of Polish and Palestinian students (*p* < 0.05). More Polish students wear a mask (77.4%) and wash hands (90.1%) compared to students from Palestine, where this percentage is 68% and 82.5%, respectively. However, in terms of physical distancing, 44.3% of the Palestinian respondents maintained it. For comparison, this was only true of 29.5% of Polish students. Figure 9 presents the results of students from Palestine and Poland on compliance with epidemic restrictions.

## 4. Discussion

This study aimed to determine the feelings of Polish and Palestinian students after receiving vaccinations against COVID-19. The obtained results indicate that respondents from Poland are optimistic about the applied vaccines and believe that this is an effective tool in the fight against the pandemic. The opposite opinion is found only among 9.3% of Polish respondents. Among the Palestinian students, 25% of respondents approach the vaccinations with hesitance and believe that they will not bring the expected effect. Taking into account all participants of the research, it should be noted that nearly half believe that vaccinations are effective. Other studies on the effectiveness of vaccines carried out in various places of Palestine, indicate a low acceptance of vaccinations and at the same time indicate good knowledge about preventive measures to avoid the spread of infection [26]. The greatest resistance to vaccinations is found among health care professionals [27].

Analyzing the obtained data on the effects of vaccination, respondents from the University of Bethlehem to a large extent are afraid of the negative effects of vaccination (57.8%), and there is less concern among Polish students (31.9%). At the same time, women from Palestine (63%) and Poland (34.2%) are more concerned, despite the fact that more than half of women from both countries positively evaluate the vaccine adopted. The reluctance of the Palestinian population for vaccines, as other studies show in this respect, results from false information provided in social media and lack of confidence in the vaccines purchased by the government [28].

It should be emphasized here that vaccines in Palestine were applied later than in other parts of the world, and in the University of Bethlehem, the administration of vaccines started in June 2021. Social awareness and prophylactic behaviors at that time received a lot of controversy, which confirms other studies in this respect [29]. The consequence of the situation is unconventional social behaviors in the mental sphere that revealed that many inhabitants from the Gaza Strip were dissatisfied with the campaigns and psychological care initiatives [30]. The obtained data from these studies show that 66.7% of men from Poland and 34.2% from Palestine perceive vaccination positively and are not afraid of the negative impacts of the preparations. Half of all participants are satisfied with the vaccinations. The opposite is true of 24% of students from the University of Bethlehem and almost 9% from Poland. The above opinions of Palestinian respondents correspond to other studies that show that the overall intention to receive COVID-19 vaccines was the highest among students who derived information about vaccinations from reliable scientific sources (63%) [31]. Other reports from research carried out in six Southeast Asian countries show the optimistic perception of the effectiveness of COVID-19 vaccines and the desire to receive them. However, almost half of the participants of this research express vaccine hesitance. The fluctuation is related to socio-economic factors and varies depending on the country [32]. The results from other studies indicate positive predictors of intentions. These are: social responsibility for others, the desire to adopt a vaccine and free time after vaccination. The predictors were the fear around the safety of the vaccine sand incredible information in the media [33].

The collected data from the conducted research answer the question if the approved vaccinations will stop the pandemic. Respondents from Poland are more optimistic in this matter, because 23.8% believe that vaccines are an effective tool against the pandemic, which is true of only 11.1% of the Palestinian population. Almost 44% of all research participants agree with this thesis, and the opposite opinion is true of 16% of Palestinians and 10% of Poles. Based on the collected data, there was a real motivation to accept vaccination. The adoption of vaccines was declared by 80% of respondents from Poland and almost 60% from the Palestinian area as an independent decision. A total of 16% of respondents from Bethlehem and almost 7% from Poland cited the influence of family. Other studies show that depression and the ability to infect relatives significantly affect the decision to adopt vaccination [34]. These studies have allowed collecting information on COVID-19. There was an attentive response among almost 88% of Polish respondents and nearly 77% of Palestinian.

The data obtained also show COVID-19 mortality among those close to the respondents. Respondents answered that 28% died on the Polish side and half on the Palestinian side. Reports from other studies show a low mortality rate among the Palestinian population, and more often among men [35]. The pandemic reduced interpersonal contacts, which is why it is in not surprising that approximately 80% of respondents cited accepting the vaccine gives them the desire to participate in public life, meetings and visit pubs, restaurants, cinemas, and theaters as their first motivation. Free traveling is the second motivation most often given by Polish students (75%), and fear of infection by Palestinians (72.9%). Reports from other studies from Palestine show that the greatest motivation during the pandemic was loneliness and lack of contact with family and friends [36]. In addition, as other studies indicate, among the Palestinian population, COVID-19 caused an increase in food consumption and generated a sedentary lifestyle [37]. Based on the collected data, the desire to work is one of the most frequently given motivations for vaccination by Palestinian research participants (55.7%), and less by Polish respondents (30.7%).

The results from other studies indicate that ambiguous laws of the Palestinian labor law were the main factor that allowed employers to use employees under the guise of the pandemic [38] while the fear of illnesses motivated more Palestinian respondents, and slightly less so the Polish. From the collected data, it follows that the most common side effect in the entire population was pain in the injection site. This is what 91% of students from the University of Bethlehem and 88% from Poland mentioned. Other most experienced ailments after accepting the vaccination were: headaches, pain in muscles and joints, chills and redness of the skin in the place of application of the vaccine. Most often, the above ailments were indicated by the respondents from Palestine, and more often women. The above data correspond to other studies in which respondents have stated that the most common symptom was pain in the injection site, asthenia, muscle pain and swelling at the place of administration of the vaccine, with women revealing more symptoms than men [39]. Based on the collected data, it can be concluded that 52 Palestinian students a doctor’s help and 34 were hospitalized after vaccination. Among Polish respondents, 11 people asked for medical attention, and 2 were hospitalized. Other studies carried out among 300 Palestinians confirm that 59 people hospitalized due to COVID-19 [40]. The above shows a large difference can be a reflection of the existential situation in Poland, where people fear admitting to welfare due to stigma. The results of respondents also show that they are at odds with their relatives. Fears of infection and disease among loved ones are more frequently expressed on the Polish side. The above-mentioned thesis is not entirely corresponding to data obtained from the research from Western Palestine, which shows the need to pay attention not only to physical health, but also mental health during COVID-19, especially among young people (more often women), people with a lower economic status and those living with a high risk of illness [41].

In terms of prevention and observance of epidemic recommendations (wearing masks, hand washing, and physical distancing behavior) the Polish students more often than Palestinians wear masks and wash their hands. Palestinian respondents maintain physical distancing more often. Similar studies carried out in the Gaza Strip reveal that most residents had good knowledge about healthy practices, but a large percentage of the population did not practice epidemic recommendations [42]. Other studies carried out among students from various Palestinian universities showed a high level of knowledge about preventive attitudes. Respondents less often pointed out wearing masks as an effective practice and many considered taking antibiotics as an effective tool in preventing COVID-19 infection [43]. Reports from research in Italy indicate that respondents were more inclined to maintain epidemic recommendations after the second dose of the vaccine. The above attitudes resulted from a higher level of confidence in expert information about the pandemic and the high incidence [44]. The results of research carried out in China are also interesting and show unconventional behaviors of people in terms of physical (social) spaces turning into discrimination and stigmatization of persons suffering from COVID-19. This generated resulted in people hiding the disease to avoid rejection by society [45]. The presented problem of the feelings of young people after receiving vaccinations does not exhaust the discussion, which from the beginning of the pandemic covers almost all areas of human life, but is part of an important reflection on the human condition in various populations of the world.

## 5. Conclusions

On the basis of the collected data, almost half of the respondents from both countries (regardless of gender) believe in the effectiveness of vaccines and more than half of the respondents are satisfied with vaccines they received. More Polish students believe in effectively stopping the pandemic by means of vaccination. Palestinian students, especially women, are more worried about the negative effects of having the vaccines. Respondents from both populations believe that undergoing vaccinations is their personal choice, but this view is more prevalent among students from Poland. Family had a strong influence on vaccination coverage among young Palestinian people. The social motivation for vaccination in both groups was the desire to participate in public life. The second factor for Poles was the ability to travel freely and the fear of infecting other people for Palestinians. The most common side effect after receiving the vaccine was pain at the site of the infection. Medical assistance was more often sought by respondents from Palestine, and to a minimal extent by respondents from Poland. The participants of the Palestinian population study were hospitalized very often while Polish to a small extent. From an existential, psychosocial and health point of view, vaccination has contributed to strengthening the vitality of a large part of the population and providing a sense of security in everyday life and social interactions with other people.

## Figures and Tables

**Table 1:** Number of respondents and average and standard deviations of their nationality, gender and age.

Group of Respondents	Polish Students		Palestinian Students	
	*N*	Age (Mean ± SD)	*N*	Age (Mean ± SD)
All respondents	332	22.43 ± 4.76	325	19.71 ± 2.29
Gender				
Women	254	22.72 ± 5.23	257	19.58 ± 1.47
Men	78	21.5 ± 2.58	68	20.24 ± 4.1
Age				
<20 years old	67	18.93 ± 0.26	173	18.55 ± 0.5
20–24 years old	218	21.75 ± 1.45	144	20.58 ± 0.87
>24 years old	47	30.6 ± 8.07	8	29.13 ± 8.51

**Table 2:** Comparison of answers to individual questions due to the nationality of respondents.

Question	Answer	Polish Students	Palestinian Students	Chi-Square Test		
				Chi-Sq	Df	*p*
How do you rate the effectiveness of the vaccine you have received?	I think it’s effective	164 (49.4%)	150 (46.2%)	33.12	2	<0.001
	I have no opinion	137 (41.3%)	92 (28.3%)			
	I don’t think it’s effective	31 (9.3%)	83 (25.5%)			
Are you worried about the negative effects of receiving the vaccine?	Yes	106 (31.9%)	188 (57.8%)	45.56	2	<0.001
	No	173 (52.1%)	111 (34.2%)			
	I have no opinion	53 (16%)	26 (8%)			
Are you satisfied with receiving the vaccine?	Yes	181 (54.5%)	170 (52.3%)	32.89	2	<0.001
	No	29 (8.7%)	78 (24%)			
	I have no opinion	122 (36.7%)	77 (23.7%)			
Do you think the adoption of the vaccine will help to contain the pandemic?	I definitely agree	79 (23.8%)	36 (11.1%)	25.61	4	<0.001
	I agree	80 (24.1%)	93 (28.6%)			
	I rather agree	65 (19.6%)	49 (15.1%)			
	Hard to say	74 (22.3%)	95 (29.2%)			
	I disagree	34 (10.2%)	52 (16%)			
Who influenced your decision to vaccinate against COVID-19?	My independent decision	268 (80.7%)	194 (59.7%)	40.19	4	<0.001
	Family	23 (6.9%)	52 (16%)			
	Doctor/nurse	3 (0.9%)	20 (6.2%)			
	Media	5 (1.5%)	9 (2.8%)			
	Other	33 (9.9%)	50 (15.3%)			
Has any of your loved ones had COVID-19 infection?	Yes	288 (87.7%)	248 (76.3%)	11.91	1	<0.001
	No	44 (13.3%)	77 (23.7%)			
Did any of your loved ones die from COVID-19?	Yes	47 (14.2%)	91 (28%)	18.96	1	<0.001
	No	285 (85.8%)	234 (72%)			

**Table 3:** Comparison of the answers of two groups of respondents to individual questions due to gender and nationality.

Question	Answer	Polish Students		Chi-Square Test			Palestinian Students		Chi-Square Test		
		Women	Men	Chi-Sq	Df	*p*	Women	Men	Chi-Sq	Df	*p*
How do you rate the effectiveness of the vaccine you have received?	I think it’s effective	123 (48.4%)	41 (52.6%)	0.559	2	0.756	120 (46.7%)	30 (44.1%)	3.638	2	0.162
	I have no opinion	106 (41.7%)	31 (39.7%)				67 (26.1%)	25 (36.8%)			
	I don’t think it’s effective	25 (9.8%)	6 (7.7%)				70 (27.2%)	13 (19.1%)			
Are you worried about the negative effects of receiving the vaccine?	Yes	97 (38.2%)	9 (11.5%)	19.59	2	<0.001	162 (63%)	26 (38.2%)	13.87	2	<0.001
	No	121 (47.6%)	52 (66.7%)				78 (30.4%)	33 (48.5%)			
	I have no opinion	36 (14.2%)	17 (21.8%)				17 (6.6%)	9 (13.2%)			
Are you satisfied with receiving the vaccine?	Yes	131 (51.6%)	50 (64.1%)	3.869	2	0.145	130 (50.6%)	40 (58.8%)	1.465	2	0.481
	No	23 (9%)	6 (7.7%)				64 (24.9%)	14 (20.6%)			
	I have no opinion	100 (39.4%)	22 (28.2%)				63 (24.5%)	14 (20.6%)			
Do you think the adoption of the vaccine will help to contain the pandemic?	I definitely agree	60 (23.6%)	19 (24.4%)	9.458	4	0.051	26 (10.1%)	10 (14.7%)	3.766	4	0.439
	I agree	67 (26.4%)	13 (16.7%)				74 (28.8%)	19 (42.6%)			
	I rather agree	41 (16.1%)	24 (30.8%)				36 (14%)	13 (19.1%)			
	Hard to say	59 (23.2%)	15 (19.2%)				76 (29.6%)	19 (27.9%)			
	I disagree	27 (10.6%)	7 (9%)				45 (17.5%)	7 (10.3%)			

**Table 4:** Comparison of the number of side effects after taking the vaccine by nationality and gender.

Variable		Answer	Side Effect after Taking the Vaccine									
			Pain at the Injection Site	Fatigue	Headache	Muscle Pain	Joint Pain	Chills	Fever	Swelling at the Injection Site	Redness at the Injection Site	Nausea
All respondents	Polish	Yes	292	205	173	182	97	142	149	101	101	51
		No	40	127	159	150	235	190	183	231	231	281
	Palestinian	Yes	296	228	253	248	210	213	216	178	173	151
		no	29	97	72	77	115	112	109	147	152	174
	Chi-square test	Chi-sq	1.706	5.166	47.721	33.54	82.67	34.275	30.981	39.848	35.144	74.602
		df	1	1	1	1	1	1	1	1	1	1
		*p*	0.191	0.023	<0.001	<0.001	<0.001	<0.00	<0.001	<0.001	<0.001	<0.001
Polish students	Women	Yes	231	167	132	148	80	105	106	77	71	31
		No	23	87	122	106	174	149	148	177	183	223
	Men	Yes	61	38	41	34	17	37	43	24	30	20
		No	17	40	37	44	61	41	35	54	48	58
	Chi-square test	Chi-sq	9.14	7.327	0.009	5.191	2.716	0.906	4.329	0.006	3.113	8.286
		df	1	1	1	1	1	1	1	1	1	1
		*p*	0.003	0.007	0.927	0.023	0.099	0.341	0.037	0.939	0.078	0.004
Palestinian students	Women	Yes	236	186	205	204	173	178	176	146	148	125
		No	21	71	52	53	84	79	81	111	109	132
	Men	Yes	60	42	48	44	37	35	40	32	25	26
		No	8	26	20	24	31	33	28	36	43	42
	Chi-square test	Chi-sq	0.854	2.89	4.755	6.40	3.91	7.535	2.251	2.064	9.365	2.339
		df	1	1	1	1	1	1	1	1	1	1
		*p*	0.355	0.089	0.029	0.011	0.047	0.006	0.134	0.151	0.002	0.126

**Table 5:** Comparison of results of answers to the question about fear of infection and disease on COVID-19 due to nationality.

Question	Mean ± SD	Median ± QD	N	Mann–Whitney Test	
				*z*-Value	*p*
Are you afraid of infection and falling ill with COVID-19?					
Polish students	2.56 ± 1.23	3 ± 0.75	332	−1.3724	0.17
Palestinian students	2.67 ± 1.19	3 ± 0.5	332		
Are you afraid of infection and falling ill with COVID-19 by someone close to you?					
Polish students	3.92 ± 1.11	4 ± 1	325	8.0309	<0.001
Palestinian students	3.1 ± 1.35	3 ± 1	325		

## Data Availability

Data available on request from the authors.
